# MEGA-ANALYSIS OF A CONSORTIUM OF FOUR CENTENARIAN STUDIES IDENTIFIES NOVEL EXTREME LONGEVITY VARIANTS

**DOI:** 10.1093/geroni/igac059.1743

**Published:** 2022-12-20

**Authors:** Harold Bae, Anastasia Gurinovich, Zeyuan Song, Anastasia Leshchyk, Mengze Li, Nir Barzilai, Thomas Perls, Paola Sebastiani

**Affiliations:** Oregon State University, Corvallis, Oregon, United States; Tufts Medical Center, Boston, Massachusetts, United States; Boston University, Boston, Massachusetts, United States; Boston University, Boston, Massachusetts, United States; Boston University, Boston, Massachusetts, United States; Albert Einstein College of Medicine, Bronz, New York, United States; Boston University, Boston, Massachusetts, United States; Tufts Medical Center, Boston, Massachusetts, United States

## Abstract

The meta-analysis of extreme longevity (EL) conducted in 2017 using a consortium of four longevity studies—the New England Centenarian Study (NECS), the Long Life Family Study (LLFS), the Southern Italian Centenarian Study (SICS), and the Longevity Gene Project (LGP)—confirmed previous associations with APOE alleles and identified additional candidate genes. The current study builds upon this work. Using an aggregated set of the four studies (mega-analysis), we sought to identify additional longevity variants. The current study includes 234 additional cases with a total of 2304 cases (median age=103, age range=[96, 119]), defined as living past the age at which less than 1% individuals from the 1900 - 1920 birth year cohorts survived and approximately 6,000 controls. Approximately 10 million genotyped and imputed SNPs that passed imputation quality score threshold of 0.7 and that had a minor allele count of 3 or greater were analyzed. We ran a mixed effects logistic model for EL adjusting for sex, top principal components, indicator for living in Denmark, indicator for living in Italy, and the genetic relationship matrix. The analysis identified 21 novel loci that are genome-wide significant, both common and rare, and these loci were not previously published. The genome-wide significant loci include genes such as ADGRL2 (p=6.46-15), HLA-DPA1 (p=1.06-8), GRK5 (p=2.01-8), TECTB (p=3.38-8), KCNQ1 (p=7.53-10), TEAD2 (p=9.53-10), and several intergenic regions. We are currently in the process of seeking replication of the top results in independent cohorts.

